# Novel Mutation in the *TSFM* Gene Causes an Early‐Onset Complex Chorea without Basal Ganglia Lesions

**DOI:** 10.1002/mdc3.13144

**Published:** 2021-02-05

**Authors:** Anne K. van Riesen, Saskia Biskup, Andrea A. Kühn, Angela M. Kaindl, Christoph van Riesen

**Affiliations:** ^1^ Charité – Universitätsmedizin Berlin, Center for Chronically Sick Children Berlin Germany; ^2^ Department of Pediatric Neurology University Medical Center Göttingen Göttingen Germany; ^3^ CeGaT GmbH, Center for Genomics and Transcriptomics Tübingen Germany; ^4^ Department of Neurology, Movement Disorder and Neuromodulation Unit Charité – Universitätsmedizin Berlin Berlin Germany; ^5^ Department of Pediatrics, Division of Neurology Charité – Universitätsmedizin Berlin Berlin Germany; ^6^ Charité – Universitätsmedizin Berlin, Institute of Cell Biology and Neurobiology Berlin Germany; ^7^ Department of Neurology University Medical Center Göttingen Göttingen Germany

**Keywords:** chorea, *TSFM*, basal ganglia

Few genes have been associated with childhood‐onset choreatic movement disorders.^1^ Here we report the first patient with a childhood‐onset chorea without structural brain abnormalities caused by compound heterozygous mutations in the mitochondrial Ts translation elongation factor gene *TSFM* (MIM*604723). TSFMs are nuclear encoded proteins that are critically involved in the translation of mitochondrial DNA. Disorders caused by mutations in the *TSFM* gene have been reported in only 16 patients so far with a wide phenotypic spectrum, ranging from fatal neonatal‐onset to moderate disease courses presenting only in early adulthood.^2^, ^3^, ^4^, ^5^, ^6^, ^7^, ^8^, ^9^, ^10^

## Case Report

**FIG. 1:**
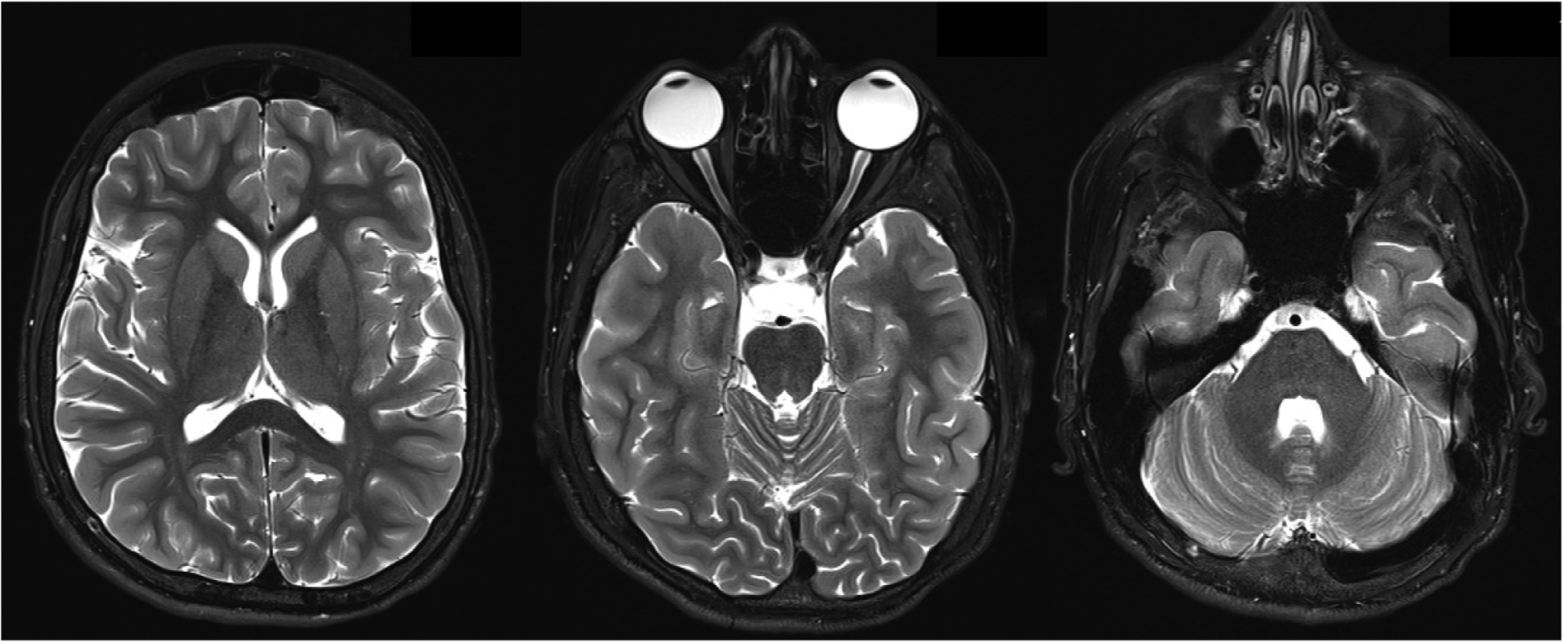
Optic nerve atrophy in patient with biallelic *TSFM* gene mutation. T2 weighted brain MRI images of the patient at the age of 13 years.

The patient is the third child of non‐consanguineous healthy parents of Crimean descent and is currently 15 years old. An older brother had died at 8 months‐of‐age and had been affected by global developmental delay, muscular hypotonia and unremitting vomiting. An older sister is healthy and normally developed. The patient was born at term without complications after an uneventful pregnancy. As a neonate he showed generalized muscular hypotonia, feeding problems and a global developmental delay. At 2 years of age he had learned to walk and speak a few words. A progressive generalized hyperkinetic movement disorder developed at the age of 2.5 years. At the first neurological examination at 5 years‐of‐age and also at the examination at 12 years of age (see Video 1), a movement disorder with prominent chorea of the face, trunk and extremities, myoclonic jerks of the neck and shoulders, intermittent dystonic postures of the neck and upper extremities and stereotypies of the hands were apparent. Furthermore, spells of involuntary upward deviation of both eyes were frequently noted. Walking was only possible with substantial assistance of one adult due to severe gait ataxia. The family reported neither diurnal changes of the severity of the movement disorder nor exacerbations upon awakening and when falling asleep. Laboratory findings were inconclusive except for elevations of serum lactate (60.7 mg/dl, norm 4.5–19.8) and pyruvate (2.5 mg/dl, norm 0.4–0.8) levels. MRIs of the brain at the age of five and 13 years were normal except for signs of progressive bilateral optic atrophy (Fig. 1). Notably, our patient showed no lesions in the basal ganglia and cerebellum in contrast to the recently reported *TSFM* case with chorea (Fig. 1).^9^ The additional diagnostic workup showed no signs of a neuropathy or a cardiomyopathy. Abnormal acoustic evoked potentials indicated a sensorineuronal hearing loss. Electron microscopic evaluation of muscle tissue demonstrated severe changes in the composition of mitochondria. Treatments with both benzodiazepines and levetirazetame were ineffective in our patient. A trial with cannabinoids, which were shown to improve chorea in one published patient recently, was denied by the parents.^9^

**Video 1:** The video shows the index patient at 12 years‐of‐age with a hyperkinetic movement disorder consisting of prominent chorea of the face, trunk and extremities, myoclonic jerks of the neck and shoulders, dystonic postures of the neck and extremities and stereotypies of the hands.

Whole exome sequence analysis demonstrated two compound heterozygous mutations in the *TSFM* gene (c.919C > T [p.Gln307*] and c.161G > A [p.Arg54Gln]; NM_001172696.1), which were confirmed through Sanger sequencing. The maternally inherited nonsense variant (c.919C > T) was published previously in 3 patients. In three of those patients this nonsense mutation was found in compound heterozygous states, twice with a missense and once with a splice site mutation, manifesting with cardiomyopathy and mild neuropathy.^2^ The paternal missense variant (c.161G > A) has not been reported before. According to published ACMG standards and guidelines the paternal variant is likely pathogenic, since the variant is at extremely low frequency in controls and is in trans with a pathogenic variant in a known autosomal recessive disease. Furthermore, missense variants are a known cause for this specific disease, also pathogenicity is predicted by various in silico analysis and the clinical phenotype and family history are in line with mitochondrial disease caused by mutations in the *TSFM* gene.

## Discussion

Disorders caused by mutations in the *TSFM* gene have only rarely been described so far with a wide phenotypic spectrum ranging from fatal neonatal‐onset syndromes to moderate disease courses with a beginning in childhood up to early adulthood even in patients carrying the same mutation.^2^, ^3^, ^4^, ^5^, ^6^, ^7^, ^8^, ^9^, ^10^ It can be speculated that the deceased older brother of our patient suffered from a more severe phenotype caused by the same compound heterozygous mutations in the TSFM gene. Unfortunately, no material was available for genetic testing. The most common symptoms reported in TSFM gene mutation carriers were cardiomyopathy and slowly progressive optic atrophy. A hyperkinetic movement disorder was found in five of the published 16 patients, however hyperkinesia did not start until late puberty or early adulthood in four patients.^2^, ^4^, ^9^, ^10^ Notably, the dominating symptom in our patient was a severe chorea since early childhood. In four of the previously reported five patients with hyperkinesia MRI was performed. Basal ganglia lesions were found in three. In contrast repeated MRI examinations in our patient showed no lesions in the basal ganglia and cerebellum. Interestingly, in the case reported by Scala et al. that suffered from ataxia and cardiomyopathy, bilateral T2 hyperintense lesions in the putamina resolved completely after several years.^6^

We therefore suggest compound heterozygous *TSFM* mutations to be considered as a relevant differential diagnosis for early‐onset chorea without structural brain abnormalities on MRI besides the recently described hyperkinetic movement disorder syndromes associated with mutations in the *ADCY5*, *PDE10a*, *PDE2a*, *NKX2‐1* or the *GNAO1* genes.^1^

## Author Roles

(1) Research Project: A. Conception, B. Organization, C. Execution; (2) Statistical Analysis: A. Design, B. Execution, C. Review and Critique; (3) Manuscript Preparation: A. Writing of the first draft, B. Review and Critique.

AKR: 1A, 1B, 1C, 3A.

SB: 1C, 2B.

AAK: 1B, 3B.

AMK: 1B, 3B.

CR: 1A, 1B, 1C, 3A.

## Disclosures

### Ethical Compliance Statement

The authors confirm that the approval of an institutional review board was not required for this work. We confirm that patient consent has been sought and allowed for this case and its publication. We confirm that we have read the Journal's position on issues involved in ethical publication and affirm that this work is consistent with those guidelines.

### Funding Sources and Conflict of Interest

No specific funding was received for this work and the authors have no conflicts to report.

### Financial Disclosures for the previous 12 months

The authors AKR, SB, AMK and CR declare that there are no additional disclosures to report. AAK: Advisory Board: Boston Scientific und Medtronic. Honoraria: Boston Scientific, Medtronic and Abbott. Lectures for Teva and Ipsen.

## References

[1] Mencacci NE , Carecchio M . Recent advances in genetics of chorea. Curr Opin Neurol 2016;29(4):486–495. 27257945 10.1097/WCO.0000000000000352 PMC4934600

[2] Ahola S , Isohanni P , Euro L , et al. Mitochondrial EFTs defects in juvenile‐onset Leigh disease, ataxia, neuropathy, and optic atrophy. Neurology 2014;83(8):743–751. 25037205 10.1212/WNL.0000000000000716 PMC4150129

[3] Calvo SE , Compton AG , Hershman SG , et al. Molecular diagnosis of infantile mitochondrial disease with targeted next‐generation sequencing. Sci Transl Med 2012;4(118):118ra110. 10.1126/scitranslmed.3003310 PMC3523805 22277967

[4] Emperador S , Bayona‐Bafaluy MP , Fernandez‐Marmiesse A , et al. Molecular‐genetic characterization and rescue of a TSFM mutation causing childhood‐onset ataxia and nonobstructive cardiomyopathy. Eur J Hum Genet 2016;25(1):153–156. 27677415 10.1038/ejhg.2016.124 PMC5159760

[5] Perli E , Pisano A , Glasgow RIC , et al. Novel compound mutations in the mitochondrial translation elongation factor (TSFM) gene cause severe cardiomyopathy with myocardial fibro‐adipose replacement. Sci Rep 2019;9(1):5108. 30911037 10.1038/s41598-019-41483-9 PMC6434145

[6] Scala M , Brigati G , Fiorillo C , et al. Novel homozygous TSFM pathogenic variant associated with encephalocardiomyopathy with sensorineural hearing loss and peculiar neuroradiologic findings. Neurogenetics 2019;20(3):165–172. 31267352 10.1007/s10048-019-00582-5

[7] Shamseldin HE , Alshammari M , Al‐Sheddi T , et al. Genomic analysis of mitochondrial diseases in a consanguineous population reveals novel candidate disease genes. J Med Genet 2012;49(4):234–241. 22499341 10.1136/jmedgenet-2012-100836

[8] Smeitink JA , Elpeleg O , Antonicka H , et al. Distinct clinical phenotypes associated with a mutation in the mitochondrial translation elongation factor EFTs. Am J Hum Genet 2006;79(5):869–877. 17033963 10.1086/508434 PMC1698578

[9] Traschutz A , Hayer SN , Bender B , Schols L , Biskup S , Synofzik M . TSFM mutations cause a complex hyperkinetic movement disorder with strong relief by cannabinoids. Parkinsonism Relat Disord 2019;60:176–178. 30297209 10.1016/j.parkreldis.2018.09.031

[10] Vedrenne V , Galmiche L , Chretien D , de Lonlay P , Munnich A , Rotig A . Mutation in the mitochondrial translation elongation factor EFTs results in severe infantile liver failure. J Hepatol 2012;56(1):294–297. 21741925 10.1016/j.jhep.2011.06.014

